# Breast and endometrial safety of micronised progesterone versus norethisterone acetate in menopausal hormone therapy (PROBES): study protocol of a double-blind randomised controlled trial

**DOI:** 10.1136/bmjopen-2023-082749

**Published:** 2024-10-23

**Authors:** Christina Lundell, Nikolaos Stergiopoulos, Liselott Blomberg, Dorina Ujvari, Ina Schuppe-Koistinen, Helena Kopp-Kallner, Stavros I Iliadis, Alkistis Skalkidou, Angelica Linden Hirschberg

**Affiliations:** 1 Karolinska Institutet, Stockholm, Sweden; 2 Karolinska Institute, Solna, Sweden; 3 Women's and Children's Health, Uppsala University, Uppsala, Sweden; 4 Uppsala University, Uppsala, Sweden

**Keywords:** Sex steroids & HRT, Randomized Controlled Trial, Community gynaecology

## Abstract

**Introduction:**

Data suggest that micronised progesterone (mP) in menopausal hormone therapy is safer for the breast than synthetic progestins, while protection of the endometrium appears to be less effective. However, comparative randomised trial data are lacking. The objective of the Progesterone Breast Endometrial Safety Study is to investigate breast and endometrial safety of mP versus norethisterone acetate (NETA) in continuous combination with oral oestrogen.

**Methods and analysis:**

This multicentre trial, conducted at three University Hospitals in Stockholm and Uppsala, Sweden, consists of two phases: part 1 focuses on breast safety and is designed as a double-blind, randomised controlled trial. 260 postmenopausal women will be randomised to 100 mg mP or 0.5 mg NETA per day in continuous combination with 1 mg oestradiol. The primary objective is to compare the treatments with respect to percentage change in mammographic breast density after 12-month treatment. Secondary outcomes are breast proliferation, endometrial histology and proliferation, bleeding pattern, gut and vaginal microbiome, hormone levels and coagulation and metabolic factors, mood, and health-related quality of life. Part 2 features an open, single-arm design to study endometrial safety of 1-year treatment with mP in continuous combination with oestradiol on endometrial pathology (hyperplasia and cancer). We will treat 260 additional women with 100 mg mP/1 mg oestradiol resulting in an endometrial safety population of 390 women. The total number of participants in part 1 and part 2 will be 520.

**Ethics and dissemination:**

The study protocol was approved by the Swedish Ethical Review Authority (2021-03033) on 29 June 2021 with amendment (2023-01480-02, protocol version 3.1) on 14 March 2023. Results of the study will be published in peer-reviewed journals and presented at scientific meetings.

**Trial registration number:**

NCT05586724.

**STRENGTHS AND LIMITATIONS OF THIS STUDY**

- This is a double-blind, multicentre randomised controlled trial investigating both breast and endometrial safety of micronised progesterone versus norethisterone acetate in continuous combination with oral oestrogen.
- Study treatment is limited to 1 year.
- Mammographic breast density will be evaluated as a surrogate marker for breast cancer and endometrial pathology as a surrogate marker for endometrial cancer.
- All mammograms and endometrial biopsies will be evaluated by assessors blinded to the patient’s identity and type of treatment.
- Another strength is that data will be collected by a digital platform.

## Introduction

 Menopausal hormone therapy (MHT) for climacteric symptoms in women started in the 1960s and reached its peak in the late 1990s. Most scientific reports from observational studies published at that time showed the beneficial effects of MHT on women’s health.^1^,^3^ However, these promising findings were thereafter overturned when the first results of the Women’s Health Initiative (WHI) randomised controlled trial were published in 2002.^4^ The trial was terminated early due to increasing rates of cardiovascular disease, breast cancer and stroke among postmenopausal women using conjugated equine oestrogen plus medroxyprogesterone acetate therapy.^5^,^7^ Despite the concerns raised for the generalisability of the WHI findings, mostly focused on older age of women (mean age 63 years) and long periods after menopause (>10 years),^8^ the number of women treated with MHT subsequently greatly diminished because of fear of risks with the treatment.^9 10^

There is a great need to find safe MHT able to control excessive endometrial stimulation from the oestrogen component without stimulatory effects on the breast by the combination of oestrogen/progestogens. Combinations of oestradiol and synthetic progestins, particularly norethisterone acetate (NETA), remain the most common types of MHT used in the Nordic countries. Long-term treatment with oestrogens in combination with synthetic progestins is associated with a small but known increased risk of breast cancer,^11^,^13^ whereas the risk of endometrial cancer with continuous combined MHT is decreased.^4 14 15^

Micronised progesterone (mP) was first introduced in the late 70s. At present, it is gaining in popularity in combination with oral or transdermal oestradiol as MHT in many European countries. Observational studies indicate a lower risk of breast cancer using mP in combination with oestrogen compared with combinations with synthetic progestins.^13 16 17^ On the other hand, observational studies indicate a higher risk of endometrial cancer with mP compared with progestins,^18^,^20^ whereas randomised trials have shown adequate endometrial safety with mP combined with oestrogen.^21^,^23^ Recent international guidelines recognise combinations with mP as potentially safer than progestogens for the breast but data from randomised trials are requested.^24 25^ There is a need to obtain increased knowledge from randomised controlled trials about the balance between breast and endometrial safety of mP versus progestins in combination with oestrogen.

The aim of this double-blind, multicentre, randomised, controlled trial is to compare the effects of 12-month treatment with mP versus NETA, both in combination with oestradiol, on breast and endometrial safety in healthy postmenopausal women. The primary outcome is mammographic breast density as this is the strongest independent risk factor for breast cancer.^26^ Endometrial safety will be evaluated as a composite measure of endometrial hyperplasia and cancer.

## Methods and analysis

### Objectives and study design

**Figure 1:**
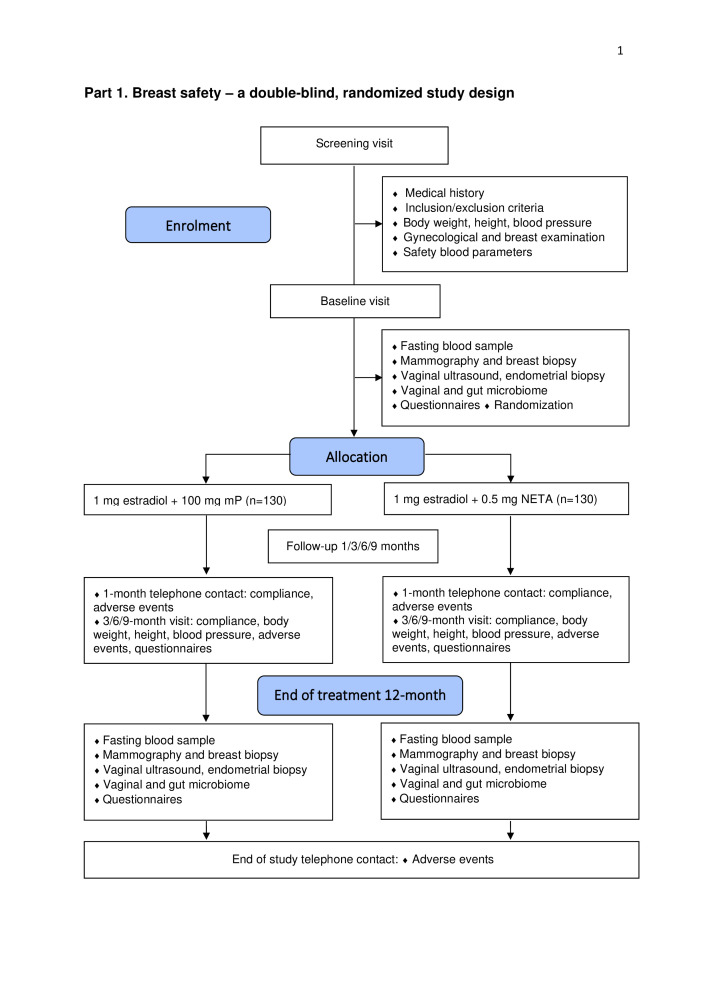
Flow diagram of part 1: breast safety—a double-blind, randomised study design. mP, micronised progesterone; NETA, norethisterone acetate.

**Figure 2:**
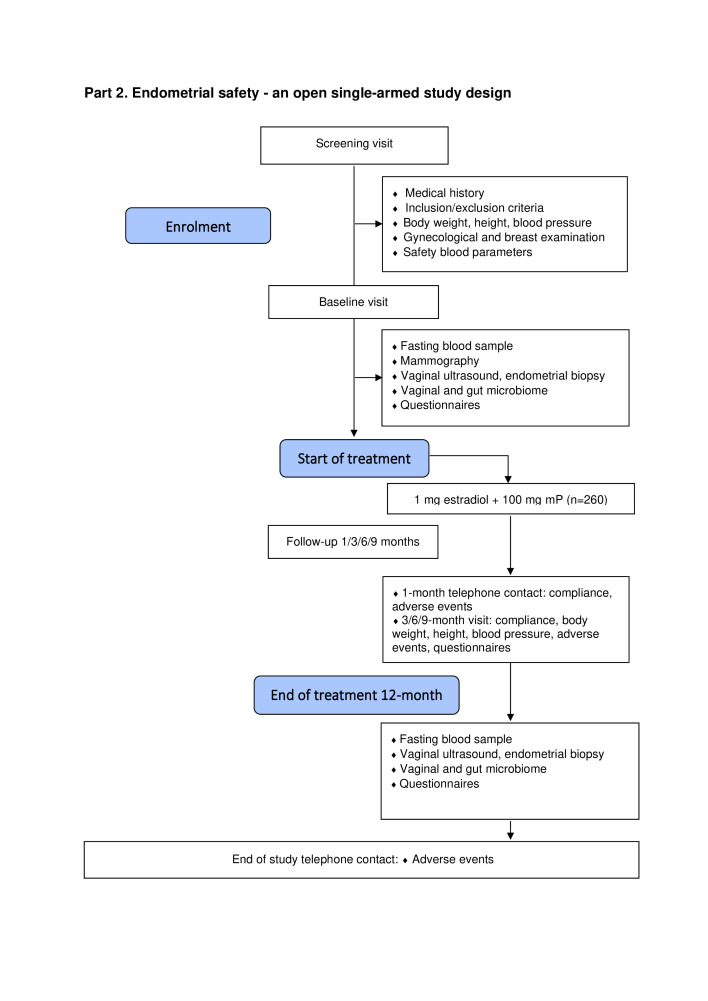
Flow diagram of part 2: endometrial safety—an open single-armed study design. mP, micronised progesterone.

This study is a double-blind, multicentre randomised controlled trial investigating both breast and endometrial safety of mP versus NETA in continuous combination with oral oestrogen (Eudract: 2021-001624-17 and ClinicalTrials.gov: NCT05586724). The trial is divided into two parts described below: part 1 is a double-blind, multicentre, randomised, controlled trial comparing the two treatments with respect to breast safety as primary objective (figure 1), and part 2 is an open, single arm to study endometrial safety of mP in continuous combination with oestradiol (figure 2).

#### Part 1: breast safety: a double-blind, randomised study design

##### Primary objective

To evaluate the effects of 12-month treatment with mP versus NETA, both in combination with oestradiol, on mammographic breast density.

##### Secondary objectives

To compare the effects of 1-year treatment with mP versus NETA, both in combination with oestradiol on the following parameters:

- Breast cell proliferation.
- Endometrial histology and proliferation.
- Endometrial thickness using ultrasound.
- Bleeding pattern.
- Gene and protein expression of growth factors and apoptosis markers in breast and endometrial tissue.
- Vaginal and gut microbiome.
- Blood levels of serum hormones, growth and metabolic factors, lipid profile, coagulations factors.
- Scores on validated questionnaires: depression and anxiety symptoms, health-related quality of life, Women’s health (menopausal symptoms).

### Part 2: endometrial safety: open, single-arm, study design

#### Primary objective

To evaluate the effect of 12-month treatment with mP in continuous combination with oestradiol on endometrial pathology (hyperplasia and cancer).

### Study population and recruitment

Healthy postmenopausal women aged 45–60 years with climacteric symptoms will be recruited from existing cohorts, women’s clinics and by advertisement in local newspapers and social media. Interested women are asked to respond by email and are subsequently contacted by telephone by a research midwife for an initial screening. Those judged to be preliminary eligible at telephone screening are sent written information about the study and invited to a screening visit for informed consent and assessment of inclusion and exclusion criteria as specified below.

When all 260 women in part 1 have been included, recruitment for part 2 will start.

### Inclusion criteria

- Healthy and naturally postmenopausal women (more than 1 year since last menstruation or follicle-stimulating hormone (FSH)>40 IE/L) with climacteric symptoms (sweating, hot flush and/or sleep problems) that adversely affect the quality of life.
- Age 45–60 years.
- Body mass index >19 kg/m^2^ and ≤32 kg/m^2^.
- Intact uterus.
- In case of previous MHT use, washout 8 weeks for oral MHT and 4 weeks for transdermal MHT or local oestrogen treatment before screening.
- Written informed consent.

### Exclusion criteria

- A history or risk factors for breast cancer, breast cancer in situ or abnormal mammogram at baseline as assessed clinically by a radiology expert.
- A history or risk factors for endometrial cancer or hyperplasia or abnormal/proliferative endometrial biopsy at baseline.
- Vaginal bleeding.
- Any concomitant medical treatment except for well-controlled hypertension, non-insulin-treated type 2 diabetes, asthma and hypothyroidism.
- History or the presence of or risk factor for cardiovascular disease including thromboembolic disorder or cerebrovascular disease.
- History or the presence of liver and gallbladder disease, familial hyperlipidaemia, epilepsy or classical migraine with aura.
- A history or the presence of clinically significant depression or other psychiatric disorder that might in any way compromise the performance of the trial or undermine its scientific validity.
- Porphyria, systemic lupus erythematosus and otosclerosis.
- Current use of MHT or local oestrogen treatment.
- Alcohol and/or drug abuse.
- Clinically significant findings on physical and/or gynaecological examination at baseline.
- Hypersensitivity to any of the study treatments.

### Interventions

#### Part 1: breast safety: a double-blind, randomised study design

Two parallel groups of postmenopausal women will be randomised to 12-month treatment of:

Group A: Capsule 100 mg mP (Utrogestan) orally per day in continuous combination with 1 mg encapsulated oestradiol (Estrofem).

Group B: Capsule 0.5 mg NETA/1 mg oestradiol (Activelle) orally per day (encapsulated and identical to Estrofem) and one matched placebo capsule to Utrogestan.

### Part 2: endometrial safety: open, single-arm, study design

In this open part of the study, all women will take a capsule of 100 mg mP (Utrogestan) orally per day in continuous combination with tablet 1 mg oestradiol (Estrofem) for 12 months.

### Randomisation

In part 1, patients will be randomised (in a 1:1 ratio) to 12 months of study treatment. The randomisation list will be created electronically using random permuted blocks with a random block size of a maximum of 6. This will be carried out by Apotek Produktion & Laboratorier and kept at the Karolinska University Hospital pharmacy. All personnel involved directly in the study shall be unaware of the treatment assigned until study closure and completion of analyses. Mammography examinations will be performed at different centres; however, evaluation of mammograms will be performed centrally by independent specialists blinded to treatment. Collection of breast biopsies will be performed only at Karolinska University Hospital. As no effect of centres is assumed, randomisation will be pooled over the centres.

### Study assessments and procedures

The study will be performed at three university hospitals in Sweden: Karolinska University Hospital, Stockholm, Danderyd Hospital, Stockholm and Uppsala University Hospital, Uppsala. The study assessment and procedures are presented in table 1.

**Table 1:** Study visit procedures

Test or procedures	Screening visit	Baseline visit	1-month contact	3-month visit	6-month visit	9-month visit	End of study visit	13-month contact
Informed consent	x							
Medical history	x							
Inclusion criteria	x	x						
Exclusion criteria	x	x						
Blood sample	x*	x†					x†	
Physical examination‡	x	x					x	
Gynaecological examination§	x	x					x	
Endometrial biopsy		x					x	
Mammography¶		x					x¶	
Breast biopsy**		x					x**	
Microbiota sample		x					x	
Questionnaire††		x		x	x	x	x	
Randomisation		x						
Compliance			x	x	x	x	x	
Vital signs‡‡				x	x	x	x	
Adverse events			x	x	x	x	x	x

* Safety parameters including hematologyhaematology, lipids, liver enzymes.

† Fasting blood sample for analysis of hormones, metabolic factors, and coagulation factors.

‡ Body weight, blood pressure, breast examination, cor, pulm.

§ Including vaginal ultrasound.

¶ A second mammography only in Ppart 1.

** Breast biopsies only in P part 1.

†† Depression and anxiety symptoms (PHQ-9, HADS), Hhealth-related quality of life (PGWB), Women’s Health Questionnaire ().

‡‡ Including bodyweight, blood pressure.

HADSHospital Anxiety and Depression ScalePGWBPsychological General Well-Being IndexPHQ-9Patient Health Questionnaire-9

#### Screening visit

At the screening visit, the woman will be informed orally and in writing about the study and will have the opportunity to ask questions. If she agrees to participate, she will sign an informed consent form (online supplemental material). The patient will be given a copy of the fully signed consent signed by herself and the investigator/coinvestigator. Thereafter, she will undergo a physical examination, gynaecological assessment and blood sample for measurement of safety parameters including haematology, lipids and liver enzymes.

#### Baseline visit

At the baseline visit, the investigator will review inclusion/exclusion criteria again to determine eligibility to participate in the study. The visit is in the morning for collection of a fasting blood sample for analysis of hormones, metabolic factors and coagulation factors. Furthermore, there will be measurements of body weight and blood pressure, gynaecological examination including vaginal ultrasound, endometrial biopsy and microbiome samples from vagina and rectum, mammography, and breast biopsy (Stockholm-centres only) (part 1), and self-assessment of depression, anxiety, quality of life and menopausal symptoms. Eligible women will be randomised (part 1) to study treatment and start their study medication next day and asked to register any bleeding in their bleeding diary.

#### One-month telephone contact

One month after start of treatment, a research midwife will have a telephone contact with the patient for follow-up of symptoms, compliance and possible adverse events.

#### Three, 6 and 9 months visit

Physical visits will be performed at 3, 6 and 9 months for assessment of vital signs, body weight and blood pressure, compliance, drug tablet return and adverse events. Furthermore, the participants will fill in the same questionnaires as at baseline.

#### End of treatment (12 months)

At end of treatment, the same investigations as at baseline will be performed with the exception of a second mammography, which will only be performed in part 1.

#### Thirteen-month telephone contact

The study ends with a telephone contact with a research midwife.

### Discontinuing study treatment

Subjects may be discontinued from study for any of the following reasons:

1. Development of exclusion criteria during the course of the study.
2. Serious adverse event.
3. Significant protocol violation.
4. Unacceptable toxicity.
5. Failure to comply with study evaluations/visits which would prevent evaluation of drug compliance and supply.
6. Other conditions for which, in the investigator’s opinion, it is in the subject’s best interest to be withdrawn from the study.

When a subject withdraws from the study, the reason for the withdrawal shall be recorded. Any subject who prematurely terminates participation and has received at least one dose of study drug will undergo end-of-study procedures. If study participation is terminated due to an adverse event possibly related to the study drug or study examinations, the subject must be followed up according to the medical judgement of the investigator.

### Breaking the code

Code breaking will occur after completion of the trial unless it is necessary to break the code due to safety reasons. In the case of a serious adverse event or suspected unexpected serious adverse reaction, only the principal investigator (and the sponsor) has the right to unblind the given treatment to a patient in case of emergency. One set of emergency code envelopes will be kept by the sponsor at the coordinating centre (Karolinska University Hospital) in a locked facility. All recruited participants will be given contact details to the trial team including emergency contact 24 hours a day, 7 days per week. Emergency unblinding can occur at any time. Details of the unblinding will be documented.

### Outcomes

#### Mammographic breast density: primary outcome in part 1

Full-field digital mammography at baseline and after 12 months of treatment comprises mediolateral oblique and craniocaudal views of both breasts. Abnormalities will be evaluated on both views of each breast. In addition to visual judgement, a computer-based quantitative assessment will be performed. All mammograms will be pseudonymised so that the assessor will be unaware of the patient’s identity and type of treatment. The dense area on the right craniocaudal view image will be measured by using a commercial computer-assisted programme. A computer then records the number of pixels that fall within the defined areas according to the methodology described and used by us previously.^27^,^30^ Percentage change in mammographic density will be evaluated and compared between groups.

#### Breast cell proliferation: secondary outcome in part 1

Core needle breast biopsy from the upper lateral quadrant of the right breast will be taken after local anaesthesia at baseline and after 12 months of treatment. Ultrasound is used to identify areas with fibroglandular tissue. All women will be biopsied at the Mammography unit, Karolinska University Hospital. Tissue samples are paraffin-embedded and sectioned at 5 µm until analysis. Slides will be stained for the proliferation marker Ki-67 and other markers related to proliferation and apoptosis (caspase-3, p53 and bcl-2), and steroid hormone receptors. In addition to standard immunohistochemistry, a quantitative image analysis will be performed using a Leica microscope and Sony video camera connected to a computer with an image analysis system (Leica Imaging System, Cambridge, UK).^27 31^ Percentage change in breast cell proliferation will be evaluated and compared between the groups.

#### Endometrial assessment: secondary outcome in part 1 and primary outcome in part 2

The bleeding pattern will be registered by the patient throughout the study by a digital mobile application. Gynaecological examination including transvaginal ultrasound to determine endometrial thickness, as well as endometrial aspiration biopsy, will be collected at baseline and after 12 months of treatment. In part 1, endometrial histology and changes in proliferation markers, such as Ki-67, growth factors and markers related to apoptosis, will be compared between the randomised groups.^32^,^34^In part 2, the incidence of endometrial pathology (hyperplasia or cancer) will be examined in all participants allocated to 12 months of daily treatment with 100 mg mP in combination with 1 mg oestradiol. Endometrial histopathology will be evaluated according to a structured histological classification by two pathologists at the Department of Pathology, Karolinska University Hospital blinded to treatment and time of biopsy. In case of disagreement in interpretation, a third pathologist, also blinded, should be called on to make the final determination. The following histological classification will be used in both part 1 and part 2: no/insufficient tissue for evaluation, atrophic endometrium, inactive endometrium, proliferative endometrium, secretory endometrium, menstrual type, simple hyperplasia, complex hyperplasia, atypical hyperplasia and carcinoma.^32^

#### Vaginal and gut microbiome: secondary outcome

Vaginal and rectal microbiome will be characterised based on shotgun metagenomic sequencing and metabolomic profiling. DNA extraction will be performed with standardised and automated pipelines for vaginal^35^ and rectal samples.^36^ Taxonomical profiling will be based on metagenomic sequencing to identify important organisms on species/strain level as well as to characterise their functional information such as enzymes involved in metabolic pathways and antibiotic resistance. Untargeted metabolomic analysis of vaginal and rectal samples will be carried out using ultrahigh performance liquid chromatography-tandem mass spectroscopy.^37^ The absolute and relative abundance of different microbial species and diversity in the women before and after 12-month treatment will be evaluated and compared between groups.

#### Serum levels of hormones, growth and metabolic factors, lipid profile and coagulation factors

Venous blood samples in a fasting state will be drawn at baseline and after 12 months of treatment for determination of circulating hormones and growth factors (FSH, luteinising hormone, oestradiol, progesterone, testosterone, sex hormone-binding globulin and insulin-like growth factor I), the lipid profile (total cholesterol, high-density lipoprotein, low-density lipoprotein and triglycerides) and coagulation factors (antithrombin, factor V Leiden, factor II mutation, cardiolipin-ab, lupus anticoagulant, protein C activity, protein S free, activated partial thromboplastin time, fibrinogen, prothrombin complex and thrombocytes). Correlations between serum markers and primary outcome variables will be performed.

#### Depression, anxiety, quality of life and menopausal symptoms

Validated self-assessment questionnaires will be used for screening of mood disorders, quality of life and menopausal symptoms. The Patient Health Questionnaire (PHQ-9) is a tool for screening, diagnosing and measuring the severity of depression.^38^ The Hospital Anxiety and Depression Scale (HADS) is an instrument for detecting states of depression and anxiety in the setting of a hospital or medical outpatient clinic.^39^ Health-related quality of life is measured using the Psychological General Well-Being Index (PGWB).^40^ The Women’s Health Questionnaire (WHQ) measures menopausal symptoms.^41^ Changes in scores will be compared between the groups.

### Sample size calculation

#### Mammographic breast density: primary outcome in part 1

Based on data from the WHI study, it was shown that an increase in mammographic density by 1% during combined MHT corresponded to a 3% increase in risk of breast cancer.^42^ We estimated that a difference of at least 30% in treatment response between the groups would be clinically relevant. A power analysis based on our previous results,^27^,^30^ and assuming a 35% difference in treatment response, revealed that a patient material of 91 women/group would be sufficient to detect a significant difference in mammographic breast density between the groups at the 5% level (two sided) with 80% power. Considering the estimated rate of discontinuation and incomplete data (~30%), the target sample for this part of the study would be 260 patients.

#### Endometrial pathology: primary outcome in part 2

The reported 1-year background incidence rate for endometrial hyperplasia/cancer in postmenopausal women treated with authorised oestrogen/progestogen combinations is approximately 0%–1%.^18 19^ With a sample size of 300 women in the treatment arm mP+E, two or less women with serious adverse endometrial outcomes would result in an annual incidence of 0.67% or less with an upper bound of the one-sided 95% CI of 2.08% or less. Considering the estimated rate of discontinuation and incomplete data (~30%), the target sample for this part should be 390 patients, 130 women randomised to mP from part 1 and another 260 women from part 2.

The total number of patients in part 1 (n=260) and part 2 (n=260) will be 520.

### Data statement section

A digital platform is used for data entry by the patients (questionnaires and eventual bleeding) and the study personnel. All patient data are encoded according to a code list, which is only available to the researchers in the project. The results are processed after the deidentification.

#### Statistical analysis

Analyses of primary and secondary outcomes will be based on an intention-to-treat (ITT) population (all subjects who have received at least one dose of study drug with a baseline evaluation meeting the entry criteria), as well as per-protocol population (subjects who have completed the study without major protocol deviations). The safety analysis population will comprise all patients who received at least one dose of the study drug.

The primary outcome variable mammographic breast density (part 1), expressed as percentage, will be compared between and within groups using the mixed model procedure with subjects as a random factor, and treatment (mP+E and NETA+E), time (baseline and exit) and treatment×time as fixed factors. Differences between groups will be evaluated by the interaction treatment×time, and differences within groups by the factor time, by holding the other factor (treatment) fixed.

Incidence of endometrial pathology (hyperplasia or cancer) in the mP+E group as endometrial safety (part 2) and based on histopathology will be expressed as incidence rate and 95% CI, in line with the European Medicines Agency guidelines.^43^

The secondary outcome variables (breast cell proliferation, endometrial cell proliferation, endometrial thickness using ultrasound, gene and protein expression of growth factors and apoptosis markers in breast and endometrial tissue, vaginal and gut microbiome, blood levels of serum hormones, growth and metabolic factors, lipid profile, and coagulations factors) will be analysed using the mixed model procedure with the same factors as above. In case of positively skewed distributions of these variables, log transformation will be performed prior to the analysis.

The Mann-Whitney U test will be used to compare the groups with respect to the changes in the PHQ-9, HADS, PGWB and WHQ and the Wilcoxon matched pairs test for changes in these variables within the groups.

If missing data occur in the ITT analysis, the method last observation carried forward (LOCF) will be used. Assuming missing data occur at random, patients with missing data can be included in the mixed model analysis, otherwise, we use the LOCF procedure. Correlations will be assessed by Spearman’s rank correlation test. A p<0.05 will be considered significant.

### Patient and public involvement

This project was initiated in collaboration with representatives of the Swedish Society of Obstetrics and Gynecology, due to a dramatically increased licensing prescription of mP for MHT use at the request of patients. Still, there is no approved mP product for the indication MHT in Sweden. The study is promoted by Swedish organisations for women’s health and menopause, such as the 1.6 Million Club 1,6 &2,6 miljonerklubben - För kvinnor och hälsa and ‘Klimakteriepodden’ (http://www.klimakteriepodden.se/). Collaboration with these organisations has opened for patient and public involvement of importance for the development of this protocol. The study has been highlighted in social and public media many times.

### Ethics and dissemination

The study was registered at ClinicalTrials.gov: NCT05586724 on 9 October 2022 and was approved by the Swedish Ethical Review Authority (2021-03033) on 29 June 2021 and by the Swedish Medical Product Agency (Eudract: 2021-001624-17) on 21 July 2021. The first participant was enrolled on 15 March 2022. The study initially received approval to collect blood samples but later applied for permission (2023-01480-02) to collect additional blood samples and obtain approval for microbiome sample collection, which was only conducted after approval on 24 March 2023. The trial will be conducted in accordance with the ethical principles that have their origin in the Declaration of Helsinki, ICH(the International Council for Harmonisation of Technical Requirements for Pharmaceuticals for Human Use) GCP (Guideline for Good Clinical Practice) and applicable local regulatory requirements. MHT has well-known potential risks and side effects. Women with known contraindications to MHT will not be included in the study. Before starting treatment, breast pathology is excluded by mammography and endometrial pathology by endometrial biopsy. All examinations and samplings are performed according to clinical routine and documented in the patient’s medical record. Vital signs, blood pressure, heart rate and body weight are followed throughout the study. The result of the study will be published in peer-reviewed journals and presented at scientific meetings, as well as communicated via social media and relevant patient organisations.

## Discussion

Approximately one-third of all women during menopausal transition have moderate to severe climacteric symptoms and wish treatment because of the considerable impact on quality of life.^10^ Meta-analysis has shown a beneficial risk profile with MHT for women 50–60 years.^44^ Still, there is a great need to find safe MHT able to control excessive endometrial stimulation by oestrogen without stimulatory effects on the breast by the combination of oestrogen/progestogen. Observational studies indicate a lower risk for breast cancer using mP combined with oestrogen but an increased risk of endometrial cancer than by standard MHT.^1316^,^20^ International guidelines request increased knowledge about safety from randomised trials.^24 25^

The Progesterone Breast Endometrial Safety Study is a unique double-blind, multicentre, randomised, controlled trial exploring the balance between the benefits and risks of progesterone versus synthetic progestins in combination with oestrogen. A limitation of the study is the use of surrogate markers for breast and endometrial cancer. However, using breast cancer and endometrial cancer as primary outcomes would require the inclusion of thousands of patients for long-term treatment, which was not considered feasible. Mammographic breast density is the strongest independent risk factor for breast cancer.^26^ As the primary objective of the randomised part of the trial (part 1), we chose to study the effects of a daily dose of 100 mg mP vs 0.5 mg NETA for continuous combination with 1 mg oestradiol on mammographic breast density in postmenopausal women with climacteric symptoms.

Endometrial hyperplasia is a strong risk factor for endometrial cancer.^45^ We will evaluate endometrial histology in both part 1 and part 2 of the trial, but the randomised part (n=260) is not powered for evaluation of endometrial hyperplasia and cancer, and therefore, this part will be comparing endometrial histology and proliferation between the two treatment groups as secondary objectives. The combination of 0.5 mg NETA/1 mg oestradiol (Activelle) is standard treatment with a well-known endometrial safety profile. In contrast, the combination of 100 mg mP/1 mg oestradiol has only been evaluated for endometrial safety in one previous study,^23^ and therefore, we found it important to study the incidence of endometrial hyperplasia and cancer of this treatment compared with the background incidence rate as a primary objective in part 2 of the trial.

A potential limitation of the study is the duration of treatment of 1 year. This could be regarded as short term, but previous studies have demonstrated that change in mammographic density by hormone treatment develops as soon as after a few weeks.^46^ Moreover, based on the estimated incidence rate of endometrial pathology (hyperplasia and cancer), 1 year should be sufficient for assessment of endometrial safety.^43^

The strengths of the study include the double-blind, multicentre, randomised design and the sufficient number of patients to assess the primary outcomes according to power calculations. Other strengths are blinded assessment of both mammographic density and endometrial pathology. Furthermore, secondary outcomes include a deeper investigation of the treatment effects on breast and endometrial tissue, as well as assessments of subjective symptoms and health-related quality of life by validated questionnaires. In addition, the treatment effect on the gut and vaginal microbiome will be studied. All data will be collected by a digital platform, which will facilitate the extraction and compilation of data.

The study is expected to provide increased knowledge about breast and endometrial safety with mP compared with progestin in continuous combination with oestrogen. The results can then be part of the basis for updated national and international guidelines for hormone treatment in menopause.

## supplementary material

10.1136/bmjopen-2023-082749 online supplemental file 1

## References

[1] Ettinger B, Friedman GD, Bush T (1996). Reduced mortality associated with long-term postmenopausal estrogen therapy. Obstet Gynecol.

[2] Grodstein F, Manson JE, Colditz GA (2000). A prospective, observational study of postmenopausal hormone therapy and primary prevention of cardiovascular disease. Ann Intern Med.

[3] LeBlanc ES, Janowsky J, Chan BK (2001). Hormone replacement therapy and cognition: systematic review and meta-analysis. JAMA.

[4] Rossouw JE, Anderson GL, Prentice RL (2002). Risks and benefits of estrogen plus progestin in healthy postmenopausal women: principal results From the Women’s Health Initiative randomized controlled trial. JAMA.

[5] Manson JE, Hsia J, Johnson KC (2003). Estrogen plus progestin and the risk of coronary heart disease. N Engl J Med.

[6] Chlebowski RT, Hendrix SL, Langer RD (2003). Influence of estrogen plus progestin on breast cancer and mammography in healthy postmenopausal women: the Women’s Health Initiative Randomized Trial. JAMA.

[7] Shumaker SA, Legault C, Rapp SR (2003). Estrogen plus progestin and the incidence of dementia and mild cognitive impairment in postmenopausal women: the Women’s Health Initiative Memory Study: a randomized controlled trial. JAMA.

[8] Clark JH (2006). A critique of Women’s Health Initiative Studies (2002-2006). Nucl Recept Signal.

[9] Hsu A, Card A, Lin SX (2009). Changes in postmenopausal hormone replacement therapy use among women with high cardiovascular risk. Am J Public Health.

[10] Lindh-Åstrand L, Hoffmann M, Hammar M (2015). Hot flushes, hormone therapy and alternative treatments: 30 years of experience from Sweden. Climacteric.

[11] Chlebowski RT, Anderson GL, Aragaki AK (2020). Association of Menopausal Hormone Therapy With Breast Cancer Incidence and Mortality During Long-term Follow-up of the Women’s Health Initiative Randomized Clinical Trials. JAMA.

[12] (2019). Type and timing of menopausal hormone therapy and breast cancer risk: individual participant meta-analysis of the worldwide epidemiological evidence. The Lancet.

[13] Yang Z, Hu Y, Zhang J (2017). Estradiol therapy and breast cancer risk in perimenopausal and postmenopausal women: a systematic review and meta-analysis. Gynecol Endocrinol.

[14] Furness S, Roberts H, Marjoribanks J (2009). Hormone therapy in postmenopausal women and risk of endometrial hyperplasia. Cochrane Database Syst Rev.

[15] Beral V, Bull D, Reeves G (2005). Endometrial cancer and hormone-replacement therapy in the Million Women Study. Lancet.

[16] Stute P, Wildt L, Neulen J (2018). The impact of micronized progesterone on breast cancer risk: a systematic review. Climacteric.

[17] Vinogradova Y, Coupland C, Hippisley-Cox J (2020). Use of hormone replacement therapy and risk of breast cancer: nested case-control studies using the QResearch and CPRD databases. BMJ.

[18] Allen NE, Tsilidis KK, Key TJ (2010). Menopausal hormone therapy and risk of endometrial carcinoma among postmenopausal women in the European Prospective Investigation Into Cancer and Nutrition. Am J Epidemiol.

[19] Fournier A, Dossus L, Mesrine S (2014). Risks of endometrial cancer associated with different hormone replacement therapies in the E3N cohort, 1992-2008. Am J Epidemiol.

[20] Tempfer CB, Hilal Z, Kern P (2020). Menopausal Hormone Therapy and Risk of Endometrial Cancer: A Systematic Review. Cancers (Basel).

[21] (1996). Effects of hormone replacement therapy on endometrial histology in postmenopausal women. The Postmenopausal Estrogen/Progestin Interventions (PEPI) Trial. The Writing Group for the PEPI Trial. JAMA.

[22] Jondet M, Maroni M, Yaneva H (2002). Comparative endometrial histology in postmenopausal women with sequential hormone replacement therapy of estradiol and, either chlormadinone acetate or micronized progesterone. Maturitas.

[23] Lobo RA, Archer DF, Kagan R (2018). A 17β-Estradiol-Progesterone Oral Capsule for Vasomotor Symptoms in Postmenopausal Women: A Randomized Controlled Trial. Obstet Gynecol.

[24] (2022). The 2022 hormone therapy position statement of The North American Menopause Society. Menopause.

[25] Lumsden MA (2016). The NICE Guideline - Menopause: diagnosis and management. Climacteric.

[26] Boyd NF, Rommens JM, Vogt K (2005). Mammographic breast density as an intermediate phenotype for breast cancer. Lancet Oncol.

[27] Hirschberg AL, Edlund M, Svane G (2007). An isopropanolic extract of black cohosh does not increase mammographic breast density or breast cell proliferation in postmenopausal women. Menopause.

[28] Hofling M, Lundström E, Azavedo E (2007). Testosterone addition during menopausal hormone therapy: effects on mammographic breast density. Climacteric.

[29] Davis SR, Hirschberg AL, Wagner LK (2009). The Effect of Transdermal Testosterone on Mammographic Density in Postmenopausal Women Not Receiving Systemic Estrogen Therapy. J Clin Endocrinol Metab.

[30] Lundström E, Hirschberg AL, Söderqvist G (2011). Digitized assessment of mammographic breast density--effects of continuous combined hormone therapy, tibolone and black cohosh compared to placebo. Maturitas.

[31] Hofling M, Hirschberg AL, Skoog L (2007). Testosterone inhibits estrogen/progestogen-induced breast cell proliferation in postmenopausal women. Menopause.

[32] Zang H, Sahlin L, Masironi B (2007). Effects of testosterone treatment on endometrial proliferation in postmenopausal women. J Clin Endocrinol Metab.

[33] Zang H, Sahlin L, Masironi B (2008). Effects of testosterone and estrogen treatment on the distribution of sex hormone receptors in the endometrium of postmenopausal women. Menopause.

[34] Paulson M, Sahlin L, Hirschberg AL (2017). Progesterone Receptors and Proliferation of the Endometrium in Obese Women With Polycystic Ovary Syndrome-A Lifestyle Intervention Study. J Clin Endocrinol Metab.

[35] Hugerth LW, Pereira M, Zha Y (2020). Assessment of In Vitro and In Silico Protocols for Sequence-Based Characterization of the Human Vaginal Microbiome. mSphere.

[36] Krog MC, Hugerth LW, Fransson E (2022). The healthy female microbiome across body sites: effect of hormonal contraceptives and the menstrual cycle. Hum Reprod.

[37] Manoharan L, Roth B, Bang C (2023). An Okinawan-Based Nordic Diet Leads to Profound Effects on Gut Microbiota and Plasma Metabolites Linked to Glucose and Lipid Metabolism. Nutrients.

[38] Spitzer RL, Williams JB, Kroenke K (2000). Validity and utility of the PRIME-MD patient health questionnaire in assessment of 3000 obstetric-gynecologic patients: the PRIME-MD Patient Health Questionnaire Obstetrics-Gynecology Study. Am J Obstet Gynecol.

[39] Bjelland I, Dahl AA, Haug TT (2002). The validity of the Hospital Anxiety and Depression Scale. J Psychosom Res.

[40] Chassany O, Dimenäs E, Dubois D, Fayol-Paget C, Lobo-Luppi L (2004). The psychological general well-being index (PGWBI) user manual.

[41] Hunter MS (2003). The Women’s Health Questionnaire (WHQ): Frequently Asked Questions (FAQ). Health Qual Life Outcomes.

[42] Byrne C, Ursin G, Martin CF (2017). Mammographic Density Change With Estrogen and Progestin Therapy and Breast Cancer Risk. J Natl Cancer Inst.

[43] (2005). EMA, chmp guideline on clnical evaluation of medicinal products for hormone replacement therapy of oestrogen deficiency symptoms in postmenopausal women.

[44] Boardman HMP, Hartley L, Eisinga A (2015). Hormone therapy for preventing cardiovascular disease in post-menopausal women. Cochrane Database Syst Rev.

[45] Reed SD, Newton KM, Clinton WL (2009). Incidence of endometrial hyperplasia. Am J Obstet Gynecol.

[46] Warren R (2004). Hormones and mammographic breast density. Maturitas.

