# Supplementary material for: Breast and endometrial safety of micronised progesterone versus norethisterone acetate in menopausal hormone therapy (PROBES): study protocol of a double-blind randomised controlled trial
Source: BMJ Open. 2024 Oct 23;14(10):e082749. doi: 10.1136/bmjopen-2023-082749 (PMC11499784; doi:10.1136/bmjopen-2023-082749)
Supplement: online supplemental file 1 [file bmjopen-14-10-s001.pdf]

Deltagarinformation

Information till forskningspersonerna

Du tillfrågas härmed om du vill deltaga i en klinisk studie med syfte att utvärdera säkerheten för bröst och livmoderslemhinna med kombinerad hormonbehandling innehållande antingen naturligt eller syntetiskt progesteron. Läs noga igenom denna information innan du bestämmer dig om du vill deltaga eller ej. Du kommer också att få muntlig information av din läkare och möjlighet att ställa frågor. Om du bestämmer dig för att deltaga, har du rätt att när som helst avbryta studien utan att behöva ange orsaken till ditt beslut och utan att det påverkar ditt framtida omhändertagande.

Vad är det för projekt och varför vill ni att jag ska delta?

Ungefär var tredje kvinna i klimakteriet lider svårt av svettningar, vallningar och sömnproblem och önskar därför någon form av behandling. Östrogenmedicinering ger mycket god symtomlindring och kan rekommenderas de flesta kvinnor som har klimakteriebesvär med försämrad livskvalitet. För att undvika oönskad stimulering av livmoderslemhinnan kombineras östrogen vanligtvis med progesteron (gulkroppshormon) i syntetisk form s.k. gestagen. Endast kvinnor som har opererat bort livmodern kan behandlas med enbart östrogen.

För kvinnor som påbörjar hormonmedicinering i nära anslutning till menopaus (sista menstruationen) har behandlingen många positiva hälsoeffekter, bland annat minskar den risken för skeletturkalkning och hjärt-kärlsjukdom samt förlänger livet. Däremot ger längre tids hormonbehandling en något ökad risk för bröstcancer. Idag vet vi att risken för bröstcancer framför allt gäller kombinationsbehandling med östrogen och gestagen, medan östrogen utan gestagen ökar risken mycket litet eller inte alls.

Många kvinnor är tveksamma till att börja med hormonbehandling av rädsla för bröstcancer. Konsekvensen kan istället bli att kvinnor lider i det tysta, provar mindre beprövade alternativ eller byter till antidepressiv medicinering. Det finns därför ett stort behov av att finna säker hormonbehandling i klimakteriet som skyddar livmoderslemhinnan men som samtidigt inte ökar risken för bröstcancer.

I Sverige är standardbehandling östrogen tillsammans med gestagen (noretisteron (NETA)), medan i andra europeiska länder används istället naturligt, så kallat mikroniserat progesteron (mP). Forskningsresultat talar för en lägre risk för bröstcancer vid användning av östrogen kombinerat med naturligt progesteron istället för med gestagen medan säkerheten för livmoderslemhinnan eventuellt kan vara lägre. I Sverige finns inte naturligt progesteron som registrerad produkt för hormonbehandling i klimakteriet men det går att förskriva på licens. Under de senaste åren har licensförskrivningen av progesteron ökat dramatiskt.

För att tillmötesgå kvinnors behov och önskemål om hormonbehandling är det viktigt att få ökad kunskap om hur naturligt progesteron i kombination med östrogen påverkar bröstet och livmoderslemhinnan jämfört med standardbehandling. Syftet med studien är att utforska balansen mellan fördelar och risker med dessa två olika hormonbehandlingar. Studien syftar också till att kartlägga mikrobiomet, dvs de mikroorganismer som bland annat befinner sig i slidan och tarmkanalen, och som är av betydelse för immunförsvaret och som skydd mot sjukdomsalstrande mikroorganismer som virus och bakterier. Studien förväntas ge ökad kunskap om både bröst- och livmodersäkerhet med östrogenbehandling i kombination med naturligt progesteron jämfört med gestagen.

Studien är godkänd av Etikprövningsmyndigheten och Läkemedelsverket. Forskningshuvudman för projektet är Karolinska Universitetssjukhuset. Med forskningshuvudman menas den organisation som är ansvarig för studien.

Hur går studien till?

Studien är uppdelad i två delar, del 1 och del 2. I del 1 är huvudsyftet att undersöka bröstsäkerhet medan del 2 är inriktat på livmodersäkerhet. Du kommer att erbjudas att ingå i antingen del 1 eller 2 beroende på hur många kvinnor som redan har inkluderats.

Om du önskar att delta i studien kommer du att besöka kliniken vid 6 tillfällen under en drygt 12 månaders period. Dessutom ingår att besöka en mammografienhet för alla deltagare.

I del 1 kommer du att slumpmässigt lottas till ett av två behandlingsalternativ och varken du eller studiepersonalen vet vilken behandling du får:

Grupp A: 1 kapsel Utrogestan® (mP) 100 mg dagligen samt 1 kapsel Estrofem® 1 mg dagligen

Grupp B: 1 kapsel NETA 0,5 mg dagligen kombinerat med östradiol 1 mg dagligen (Activelle®) samt 1 placebo kapsel

I del 2 får alla deltagare 1 kapsel Utrogestan® (mP) 100 mg dagligen samt 1 tablett Estrofem® 1 mg dagligen

Behandlingen pågår i 12 månader.

Del 1

Besök 1: Screeningbesök. Du får muntlig och skriftlig information om studien samt frågor om tidigare och aktuella sjukdomar. Vikt, längd och blodtryck kontrolleras. Blodprov tas (ca 15 ml) för säkerhetsparametrar. Gynekologisk och klinisk

bröstundersökning genomförs. Om du passar för studien och vill vara med, får du skriva under ett patientsamtycke. Besöket beräknas ta ca 60 minuter.

Besök 2: Studiestart. Blodprov (ca 55 ml) tas i fastande tillstånd för analys av blodfetter, hormoner, metabola faktorer och koagulationsfaktorer, vikt och blodtryck kontrolleras. Gynekologisk undersökning inklusive vaginalt ultraljud och biopsi från livmoderslemhinnan samt mikrobiella prov från slidan och ändtarmsöppningen tages. Provtagningen kan medföra visst obehag och i ytterst sällsynta fall infektion. Du får besvara frågeformulär om depression, ångest, livskvalitet, klimakteriesymtom och mikrobiom. Du erhåller även en dagbok för registrering av blödningar. Mammografi och bröstbiopsi genomförs enligt följande:

Mammografi görs på mammografiavdelningen och kan orsaka lätt smärta i brösten vid själva undersökningen då brösten komprimeras. Kompression av brösten görs för att minska stråldosen vid undersökningen och för att få bättre kvalité på själva mammografibilden. Effekten på mammografisk brösttäthet kommer att mätas.

Punktion av brösten är sedan många år en rutinmetod vid utredning av olika knutor i brösten. Vävnadsprovtagningen av bröstet görs av erfaren läkare med en nål efter lokalbedövning. Vid själva insticket i bröstet kan möjligen ett kortare obehag kännas och någon gång kan ett blåmärke uppstå.

Därefter sker randomisering till ett av behandlingsalternativen, se ovan. Sammanlagt beräknas dessa undersökningar ta ca 2 timmar. I vissa fall kan mammografi och punktion göras vid ett annat tillfälle än övriga undersökningar.

Telefonkontakt efter 1 månad: Du blir uppringd och får frågor om klimakteriesymtom samt eventuella biverkningar. Samtalet beräknas ta ca 15 minuter.

Besök 3, 4 och 5: Uppföljande besök efter 3, 6 och 9 månaders behandling: Du träffar forskningsbarnmorska som kontrollerar hur du mår, blodtryck och eventuella biverkningar inklusive blödningar. Du får också besvara frågeformulär om depression, ångest, livskvalitet och klimakteriesymtom. Tidsåtgång ca 60 minuter.

Besök 6: Avslutande besök efter 12 månaders behandling. Blodprov (ca 55 ml) tas i fastande tillstånd, vikt och blodtryck kontrolleras. Gynekologisk undersökning inklusive vaginalt ultraljud och biopsi från livmoderslemhinnan samt mikrobiella prov från slidan och ändtarmsöppningen tages. Du genomgår mammografi och bröstbiopsi enligt ovan. Du får besvara frågeformulär om depression, ångest, livskvalitet, klimakteriesymtom och mikrobiom. Undersökningarna beräknas ta totalt ca 2 timmar. I vissa fall kan mammografi och punktion göras vid ett annat tillfälle än övriga undersökningar.

Telefonkontakt: Avslutande telefonkontakt efter 13 månader: Kontroll av eventuella biverkningar. Samtalet beräknas ta ca 15 minuter.

Del 2

I del 2 ingår samtliga undersökningar enligt ovan inkl mikrobiella provtagningar förutom vävnadsprovtagning av bröstet samt endast mammografi vid studiestart.

Möjliga följder och risker med att delta i studien

Studien sker under läkarkontroll. Samtliga undersökningar som görs i studien är rutin och personalen är väl förtrogen med dem.

Du får en noggrann hälsoundersökning. Skulle vi finna att du har avvikande resultat kommer vi att hjälpa dig till den sjukvårdsinstans som bäst tar hand om problemet. Enklare avvikelse tas om hand av studiens läkare.

Vid all hormonbehandling kan bieffekter såsom tillfälliga underlivsblödningar, huvudvärk och bröstspänning uppstå. Vanligtvis är dessa i behandlingens början och är ofta övergående. Östrogen i tablett eller kapsel som sväljes kan ge en något ökad risk för blodpropp och vid eventuella riskfaktorer för blodpropp får man inte vara med i studien. Det finns inget stöd för att en kort behandlingstid på 12 månader ökar risken för bröstcancer men vid oklara mammografifynd eller tidigare bröstcancer får man inte delta i studien.

Mammografi ger en minimal strålbelastning med dagens moderna apparatur. För att få optimal bildkvalitet och minsta möjliga stråldos komprimeras brösten, vilket kan upplevas som ett övergående obehag. Strålskyddskommittén har godkänt stråldosen i studien.

Vi har möjlighet att erbjuda dig kostnadsersättning om 1000 kronor för varje vävnadsprovtagning, dvs totalt 4000 kronor för biopsi av livmoderslemhinna och bröstvävnad samt ersättning för eventuella reseutlägg.

Samtliga besök och läkemedel är kostnadsfria. Ditt deltagande i studien är helt frivilligt och du kan när som helst bestämma dig för att avbryta ditt deltagande i studien. Vid frågor är du välkommen att kontakta oss.

Vad händer med mina uppgifter?

Projektet kommer att samla in och registrera information om dig. Uppgifter om eventuella tidigare och pågående sjukdomar, dina besvär, laboratorieprover, röntgenbilder och behandling kommer att registreras i din journal samt i separat protokoll för bearbetning och analys inom studien. Syftet med protokollet är att sammanställa studiedata på ett korrekt och säkert sätt. Ansvarig för dina personuppgifter är Karolinska Universitetssjukhuset. Studieansvarig läkare, Angelica Lindén Hirschberg är ansvarig för att ta hand om din information och använda den på

ett säkert sätt.

Inga obehöriga parter kommer att få tillgång till protokollet. Oberoende granskare och läkemedelsmyndighet kan vid behov komma att jämföra de i studien rapporterade uppgifterna med de som finns i journalen. För att möjliggöra en kritisk granskning av forskningens resultat kommer dina data att sparas upp till 15 år efter att studien har avslutats.

Vi följer EU:s allmänna dataskyddsförordning (GDPR 2016/679) och andra etiska och juridiska riktlinjer för insamling, lagring och hantering av de uppgifter som samlas in om dig som studiedeltagare. All information om dig kommer att behandlas konfidentiellt. Du kommer att få ett unikt patientnummer, vilket betyder att dina uppgifter och prover är kodade så att din information förblir konfidentiell. Studieläkaren är ansvarig för nyckeln till dessa koder och din identitet och denna nyckel förvaras på ett säkert ställe. Ditt namn och dina personuppgifter kommer inte att kunna identifieras i den information som publiceras och/eller presenteras för allmänheten. Du har samma sekretesskydd som alla patienter i svensk sjukvård. Personuppgiftsansvarig myndighet är Region Stockholm, Box 22550, 104 22 Stockholm.

Enligt EU:s dataskyddsförordning har du rätt att kostnadsfritt få ta del av de uppgifter om dig som hanteras i studien, och vid behov få eventuella fel rättade. Du kan också begära att uppgifter om dig raderas samt att behandlingen av dina personuppgifter begränsas. Om du vill ta del av uppgifterna ska du kontakta studieansvarig läkare Angelica Lindén Hirschberg, tel 08 517 73326. Dataskyddsombud nås på tel: 08-517 734 29, e-post: lisa.gellerhed-vanduin@sll.se

Om du är missnöjd med hur dina personuppgifter behandlas har du rätt att ge in klagomål till Integritetsskyddsmyndigheten, som är tillsynsmyndighet.

Vad händer med mina prover?

Alla kodade prov som tas i studien kommer att hanteras i enlighet med svenska biobankslagen (SFS 2002:297) och kommer att förvaras i Stockholms medicinska biobank (registreringsnummer 914 hos Inspektionen för vård och omsorg (IVO)) och Karolinska Institutets Biobank (registreringsnummer 222 hos Socialstyrelsen).

Du har rätt att säga nej till att proverna sparas. Om du samtycker till att proverna sparas har du rätt att senare ångra det samtycket. Dina prover kommer i så fall att kastas eller avidentifieras. Om du vill ångra ett samtycke ska du kontakta ansvarig läkare för studien.

Proverna får bara användas på det sätt som du har gett samtycke till. Om det skulle tillkomma forskning som ännu inte är planerad, kommer etikprövningsmyndigheten att besluta om du ska tillfrågas på nytt.

Hur får jag information om resultatet av studien?

Om du önskar har du som deltagare i studien rätt att ta del av dina individuella resultat genom att ta kontakt med ansvarig studieläkare. Vid oförutsedda fynd kommer du att informeras och vi kommer att hjälpa dig till den sjukvårdsinstans som bäst tar hand om problemet. Enklare avvikelse tas om hand av studiens läkare.

Försäkring och ersättning

Genom patientförsäkringen och läkemedelsförsäkringen är du försäkrad mot skada som uppstår på grund av medverkan i studien.

Deltagandet är frivilligt

Ditt deltagande är frivilligt och du kan när som helst välja att avbryta deltagandet. Om du väljer att inte delta eller vill avbryta ditt deltagande behöver du inte uppge varför, och det kommer inte heller att påverka din framtida vård eller behandling. Om du vill avbryta ditt deltagande ska du kontakta ansvarig läkare för studien (se nedan).

Ansvariga för studien

Ansvarig läkare för studien är Professor Angelica Lindén Hirschberg, Kvinnohälsan, Karolinska Universitetssjukhuset, Solna, tel. 08-517 733 26.

E-mail: angelica.linden-hirschberg@sll.se

Om du har frågor om studien eller om din medverkan i studien kan du kontakta forskningsbarnmorska Lotta Blomberg vid Kvinnohälsan, Karolinska Universitetssjukhuset Solna.

Tel 08-517 737 82, E-post: kvinnohalsan.kk@karolinska.se

Samtycke till att delta i studien

Jag har fått muntlig och skriftlig informationen om studien och har haft möjlighet att ställa frågor. Jag får behålla den skriftliga informationen.

☐Jag samtycker till att delta i studien: Säkerhet med oralt mikroniserat progesteron jämfört med noretisteronacetat i kontinuerlig kombination med oralt östrogen som menopausal hormonbehandling - en dubbelblind randomiserad studie- PROBES-studien

☐ Jag samtycker till att uppgifter om mig behandlas på det sätt som beskrivs i forskningspersonsinformationen.

☐ Jag samtycker till att mina prover sparas i en biobank på det sätt som beskrivs i forskningspersonsinformationen.

Plats och datum	Underskrift	Namnförtydligande

Jag som studieläkare har givit fullständig information till patienten om alla aspekter av studien

Plats och datum	Underskrift	Namnförtydligande
